# Prenatal Classification and Perinatal Outcomes of Fetal Umbilical–Portal–Systemic Venous Shunts: A Tertiary Center Experience

**DOI:** 10.3390/diagnostics16060829

**Published:** 2026-03-11

**Authors:** Kubra Kurt Bilirer, Hale Özer Caltek, Tuğçe Arslanoğlu, Fırat Ersan, Hakan Erenel

**Affiliations:** 1 Perinatology Department, Başakşehir Çam and Sakura City Hospital, 34480 Istanbul, Turkey; 2 Perinatology Department, Kanuni Sultan Süleyman Hospital, 34303 Istanbul, Turkey

**Keywords:** umbilical–portal–systemic venous shunt, ductus venosus, Achiron classification, chromosomal abnormalities, perinatal outcomes

## Abstract

**Background/Objectives**: Umbilical–portal–systemic venous shunts (UPSVS) are rare fetal vascular anomalies with heterogeneous embryologic origins and variable perinatal implications. Although prenatal diagnosis has increased with advances in fetal imaging, data correlating prenatal subclassification with structural/genetic abnormalities and neonatal outcomes remain limited. **Methods**: This retrospective study included 50 fetuses prenatally diagnosed with UPSVS at a tertiary referral perinatology center between 2021 and 2025. Cases were subclassified according to the Achiron prenatal classification into Type 1 umbilical–systemic shunt (USS), Type 2 ductus venosus–systemic shunt (DVSS), Type 3a intrahepatic portosystemic shunt (IHPSS), and Type 3b extrahepatic portosystemic shunt (EHPSS). Prenatal ultrasound, Doppler, fetal echocardiography, and genetic testing (karyotype and chromosomal microarray) were analyzed. Perinatal metrics—including structural/genetic anomalies, fetal growth restriction (FGR), termination of pregnancy (TOP), and neonatal outcomes—were evaluated with postnatal verification. **Results**: The distribution of subtypes was Type 1: 28% (14/50), Type 2: 48% (24/50), Type 3a: 20% (10/50), and Type 3b: 4% (2/50). Gestational age at diagnosis was significantly higher in Type 3a compared with Type 1 and Type 2 (32.2 ± 2.4 vs. 21.1 ± 6.7 and 22.4 ± 5.8 weeks; *p* < 0.001). Structural anomalies were most frequent in Type 1 (13/14, 92.9%; *p* < 0.001), while FGR predominated in Type 3a (9/10, 90%; *p* = 0.006). Ductus venosus (DV) agenesis was universal in Type 1 (14/14) and Type 3b (2/2), absent in Type 2 (0/24), and present in 20% of Type 3a (2/10) (*p* < 0.001). Genetic abnormalities were detected in 57% of Type 1 (4/7) and 56% of Type 2 (9/16) fetuses, with trisomy 21 most prevalent in Type 2. TOP was highest in Type 1 (8/14, 57.1%; *p* < 0.001). Adverse neonatal outcomes occurred primarily in Type 1 and Type 3b (*p* < 0.001), whereas Type 2 demonstrated favorable neonatal outcomes. **Conclusions**: UPSVS subtype is strongly associated with structural/genetic anomalies, FGR, and neonatal outcomes, underscoring the importance of prenatal subclassification in prognostic assessment and counseling. Type 1 and Type 3b represent the highest—risk subgroups requiring delivery planning in tertiary centers, while Type 2 generally exhibits a benign perinatal course. The association between Type 3a and FGR highlights the need for detailed evaluation of the hepatic venous system in growth-restricted fetuses. However, interpretation of subgroup-specific associations should consider the relatively small sample size of Type 3b cases and the limited genetic testing performed in some Type 3a fetuses. Multicenter prospective studies are warranted to standardize diagnostic algorithms, optimize genetic testing strategies, and refine perinatal management.

## 1. Introduction

The fetal liver constitutes a complex circulatory network characterized by dense vascularization and the presence of both afferent and efferent venous components. Through the umbilical vein (UV) and ductus venosus (DV), which are unique to the prenatal period, oxygen-rich blood is directed to vital organs, primarily the fetal heart and brain [1].

In the fetal hepatic venous system, afferent inflow consists of oxygenated blood returning from the placenta via the UV and blood originating from the portal venous system, whereas efferent drainage is delivered into the systemic circulation through the hepatic veins (HV). The DV functions as a critical prenatal shunt that allows oxygenated umbilical venous blood to partially bypass the hepatic parenchyma and reach the inferior vena cava (IVC), subsequently entering the right atrium. Embryologic development of the DV is closely linked to the proper formation of the umbilical and vitelline venous systems, and abnormalities in this developmental process may result in fetal hepatic venous connection anomalies [2]. In some instances, aberrant embryologic development results in compensatory umbilical–portal–systemic venous shunts (UPSVS), whereas in others isolated DV agenesis without any shunting is observed, preserving hepatic microcirculation [3].

Although UPSVS anomalies are rare, advances in high-resolution ultrasound, color Doppler imaging, and 3D/STIC technology have substantially improved prenatal diagnostic rates. Historically, fetal hepatic venous anomalies were primarily described under the concept of “ductus venosus agenesis and abnormal DV connections” [4,5]. However, the recent literature has shifted its focus from the mere absence of the DV toward the identification of abnormal vascular connections within the fetal hepatic venous system, and multiple classification systems have been proposed [6,7]. Among these, the prenatal classification proposed by Achiron and Kivilevitch in 2016 [6] has gained the widest acceptance. This classification, in addition to considering the origin and drainage site of the shunt, also takes into account the organization of the hepatic venous circulation shaped by the umbilical vein–ductus venosus system, which is unique to the prenatal period. The presence of umbilicoplacental flow and ductus venosus–mediated hepatic bypass in fetal life results in a hepatic venous hemodynamic framework that differs fundamentally from the postnatal circulation. Accordingly, the Achiron classification is conceptually distinct from postnatal portosystemic shunt classifications that are primarily based on portal venous anatomy and postnatal flow dynamics. The Achiron classification stratifies UPSVS into four subtypes according to the origin and drainage site of the abnormal connection: Type 1 umbilical–systemic shunt (USS), Type 2 ductus venosus–systemic shunt (DVSS), Type 3a intrahepatic portosystemic shunt (IHPSS), and Type 3b extrahepatic portosystemic shunt (EHPSS) [6].

The primary hypothesis of the present study was that the UPSVS shunt subtype, as defined by the Achiron prenatal classification, would be associated with distinct patterns of structural and genetic anomalies as well as differing perinatal outcomes, thereby providing prognostic information relevant to prenatal counseling and delivery planning.

The aim of the present study was to describe, classify, and evaluate the perinatal outcomes of all UPSVS cases diagnosed prenatally in a high-volume tertiary referral perinatology center. In addition, we sought to contribute to the growing body of literature regarding the prenatal diagnosis of UPSVS, determine the spectrum of associated structural and/or genetic anomalies, assess postnatal outcomes, and facilitate prenatal counseling by informing parents about the expected perinatal course of fetuses with UPSVS.

## 2. Materials and Methods

This retrospective study included all fetuses diagnosed with UPSVS at the Perinatology Clinic of Başakşehir Çam and Sakura City Hospital, a tertiary referral center, between 2021 and 2025. Institutional ethics approval for this study was obtained from the Ethics Committee of Başakşehir Çam and Sakura City Hospital (Approval Code: 2025-75; KAEK/12.03.2025.75; Approval Date: 14 March 2025). The study was conducted in accordance with the ethical principles of the Declaration of Helsinki. As this was a retrospective study, all data were analyzed anonymously and institutional regulations regarding patient confidentiality were strictly followed.

Cases consisted of fetuses diagnosed during routine obstetric ultrasound examinations or referred to our center for fetal anomaly assessment. Cases with incomplete prenatal imaging datasets, absence of postnatal confirmation, or insufficient clinical records were excluded from the final analysis (*n* = 12).

All fetuses were evaluated using a high-resolution ultrasound system (Hitachi Arietta, Hitachi Medical Corporation, Tokyo, Japan) by perinatologists experienced in fetal imaging. Each case was assessed by one operator (KKB) and each case was discussed in a perinatal—genetic meeting with other perinatology specialists and diagnoses were confirmed. Due to the retrospective design of the study, formal intra- and interobserver variability analyses were not conducted. However, all vascular assessments and subclassifications were performed using standardized imaging planes and predefined anatomic landmarks, and diagnoses were confirmed by team consensus to ensure interpretative consistency.

**Figure 1:**
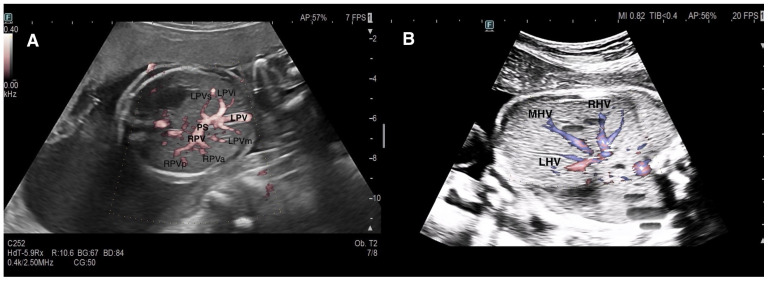
Normal fetal hepatic venous system anatomy. (**A**) Axial color Doppler view demonstrating the afferent component of the fetal hepatic venous system, including the umbilical vein (UV), portal sinus (PS), left portal vein (LPV), and right portal vein (RPV) branches. The intrahepatic portal venous system (IHPVS) is fully preserved. (**B**) Cranial axial/oblique view showing the efferent hepatic venous drainage via the right, middle, and left hepatic veins (RHV, MHV, LHV) converging toward the inferior vena cava (IVC). Abbreviations: LPVs—superior branch of the left portal vein; LPVi—inferior branch of the left portal vein; LPVm—medial branch of the left portal vein; RPVa—anterior branch of the right portal vein; RPVp—posterior branch of the right portal vein.

**Figure 2:**
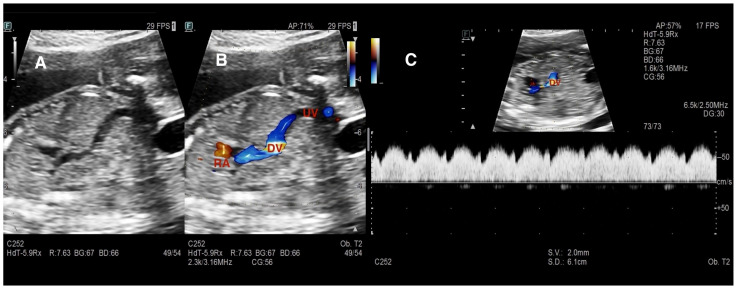
Normal ductus venosus anatomy and Doppler characteristics. (**A**) Grayscale sagittal view demonstrating the anatomic course of the ductus venosus (DV). (**B**) Color Doppler image showing the DV connecting the umbilical vein (UV) to the inferior vena cava–right atrial (RA) junction, representing normal prenatal umbilical–systemic shunting. (**C**) Spectral Doppler waveform demonstrating the characteristic triphasic DV flow pattern with forward a-wave, confirming normal hemodynamic function. Preservation of DV patency and physiologic waveform serves as a key reference for distinguishing Umbilical–portal–systemic venous shunts (UPSVS) subtypes.

In our center, evaluation of the fetal hepatic venous system begins with routine assessment of the DV. In the presence of DV abnormalities or when a hepatic venous anomaly was suspected, the hepatic venous system and its vascular components were systematically examined. Assessment included axial and sagittal planes using 2D gray-scale imaging, color Doppler, and spectral Doppler ultrasound [1]. In axial sections, Plane 1 comprised the evaluation of the afferent venous system, including the UV, left portal vein (LPV), right portal vein (RPV), main portal vein (MPV), and portal sinus (PS). In more oblique and cranial sections (Plane 2), efferent venous drainage via the hepatic veins (HV) was examined (Figure 1). Sagittal evaluation included assessment of the DV connection to the IVC at the level of the subdiaphragmatic vestibulum and confirmation of the characteristic triphasic waveform (Figure 2).

The DV was assessed for presence or absence, and if UPSVS was identified, the origin and drainage site of the shunt were documented. A detailed fetal anatomical survey and fetal echocardiography were performed as part of the examination. Cases were classified according to the Achiron prenatal UPSVS classification as follows:

Type 1: Umbilical–systemic shunt (USS)

Type 2: Ductus venosus–systemic shunt (DVSS)

Type 3: Portosystemic shunt

Type 3a: Intrahepatic portosystemic shunt (IHPSS)

Type 3b: Extrahepatic portosystemic shunt (EHPSS) [6].

**Figure 3:**
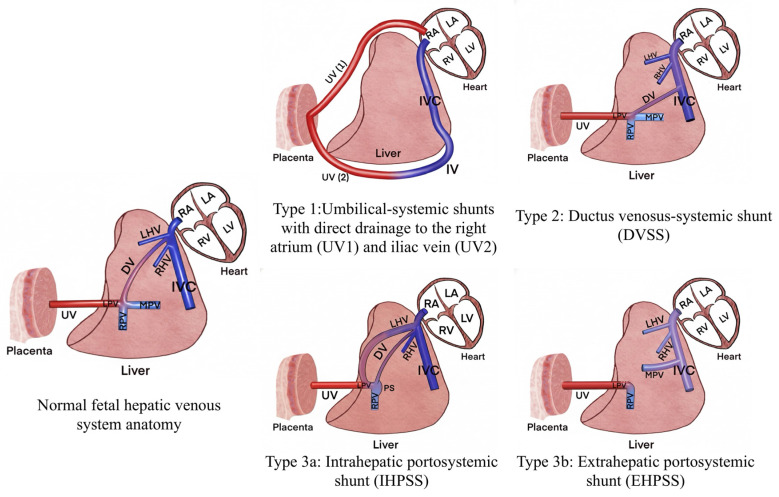
Conceptual schematic diagram of the Achiron prenatal classification of umbilical–portal–systemic venous shunts (UPSVS). The illustration summarizes the anatomic subtypes of UPSVS according to the Achiron classification, based on the origin and drainage pathway of the shunt. The normal fetal hepatic venous system anatomy is shown for reference. Type 1 USS demonstrates direct drainage of the umbilical vein into the systemic circulation, most commonly the right atrium or iliac vein, in the absence of the ductus venosus. Type 2 DVSS is characterized by an abnormal connection between the ductus venosus and systemic veins, with preservation of the intrahepatic portal venous system. Type 3a IHPSS represents intrahepatic communication between the portal venous system and systemic venous circulation, typically with preserved ductus venosus and portal venous anatomy. Type 3b EHPSS involves extrahepatic diversion of portal venous flow into systemic veins, frequently associated with partial or complete absence of the intrahepatic portal venous system. Abbreviations: UV—umbilical vein; DV—ductus venosus; LPV—left portal vein; RPV—right portal vein; MPV—main portal vein; LHV—left hepatic vein; RHV—right hepatic vein; PS—portal sinus; IVC—inferior vena cava; RA—right atrium; LA—left atrium; RV—right ventricle; LV—Left ventricle.

An explanatory schematic diagram illustrating the anatomical characteristics and drainage patterns of the Achiron UPSVS classification subtypes is provided in Figure 3.

**Figure 4:**
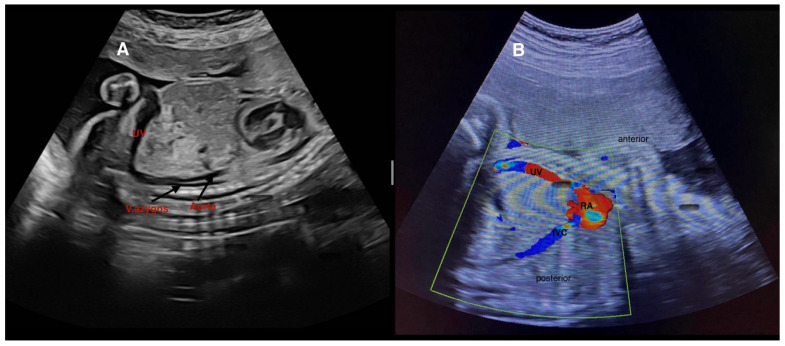
Type 1—Umbilical–systemic shunt (USS). (**A**) Grayscale image demonstrating the umbilical vein (UV) draining directly into the azygos vein (V. azygos), bypassing the hepatic parenchyma. The ductus venosus is absent, and intrahepatic portal venous perfusion is disrupted. (**B**) Color Doppler image showing the UV directly connecting to the right atrium (RA) at the level of the inferior vena cava (IVC). These findings illustrate complete extrahepatic diversion of umbilical venous flow into the systemic circulation, a hallmark feature of Type 1 USS.

**Figure 5:**
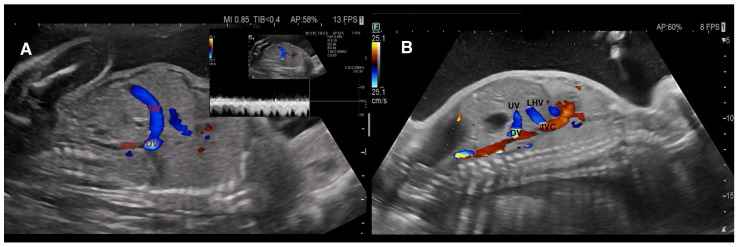
Type 2—Ductus venosus–systemic shunt (DVSS). (**A**) Color Doppler image demonstrating the ductus venosus (DV) connecting abnormally to the inferior vena cava (IVC) below the subdiaphragmatic vestibulum, following an inferior and atypical course. Spectral Doppler confirms preserved triphasic waveform. (**B**) Parallel course of the left hepatic vein (LHV) and the aberrantly oriented DV before systemic drainage. The DV is present but anomalously connected, while the intrahepatic portal venous system remains preserved—key distinguishing features of Type 2 DVSS.

**Figure 6:**
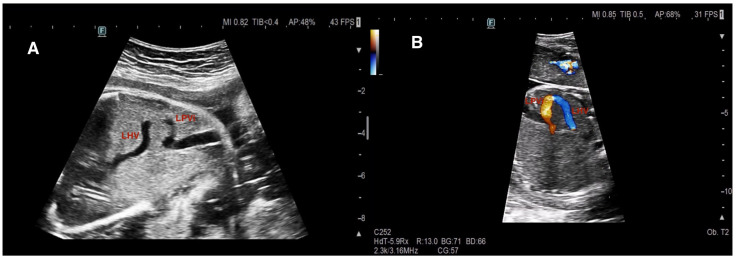
Type 3a—Intrahepatic portosystemic shunt (IHPSS). (**A**) Grayscale image demonstrating an abnormal intrahepatic vascular communication between the left portal vein (LPV) and the left hepatic vein (LHV). (**B**) Color Doppler image confirming shunt flow between the LPV and LHV. The intrahepatic portal venous system is preserved.

Representative prenatal sonographic images are presented as follows: two USS cases in Figure 4, two DVSS cases in Figure 5, and two IHPSS cases in Figure 6.

A total of 50 fetuses were retrospectively reviewed. Invasive prenatal diagnostic testing (chorionic villus sampling, amniocentesis, or cordocentesis) was recommended to all patients. When accepted, testing was performed according to gestational age, and the samples were analyzed by karyotyping and chromosomal microarray (CMA).

Fetal growth restriction (FGR) was defined according to the Delphi consensus criteria as an estimated fetal weight and/or abdominal circumference below the 3rd percentile, or below the 10th percentile in the presence of abnormal Doppler findings or growth deceleration.

Perinatal outcomes were obtained from hospital records and postnatal telephone follow-up. Delivery mode, gestational age at birth, birth weight, 1- and 5 min Apgar scores, need for phototherapy, and neonatal intensive care unit (NICU) admission were recorded. Postnatally, hepatobiliary ultrasonography, color Doppler evaluation of the portal venous system, and echocardiography were performed. Postnatal vascular imaging was used to assess portal venous anatomy and persistent shunts when applicable. Cases without accessible postnatal follow-up data were excluded from the study. After excluding secondary causes such as prematurity-related complications, respiratory disorders, and neonatal sepsis, adverse neonatal outcome was defined as either neonatal death or ≥7 days of NICU admission due to abnormalities attributable to the shunt (e.g., hyperbilirubinemia, hyperammonemia, portal hypertension, feeding difficulty, shunt-related refractory hypoglycemia, or need for surgery for associated anomalies).

Statistical analysis was performed using SPSS v.26 software (IBM Corp., Armonk, NY, USA). Continuous variables were expressed as mean ± standard deviation (SD) or median (IQR), while categorical variables were presented as number and percentage. One-way ANOVA or Kruskal–Wallis tests were used for continuous variables and chi-square or Fisher’s exact tests for categorical variables. A *p*-value < 0.05 was considered statistically significant.

**Figure 7:**
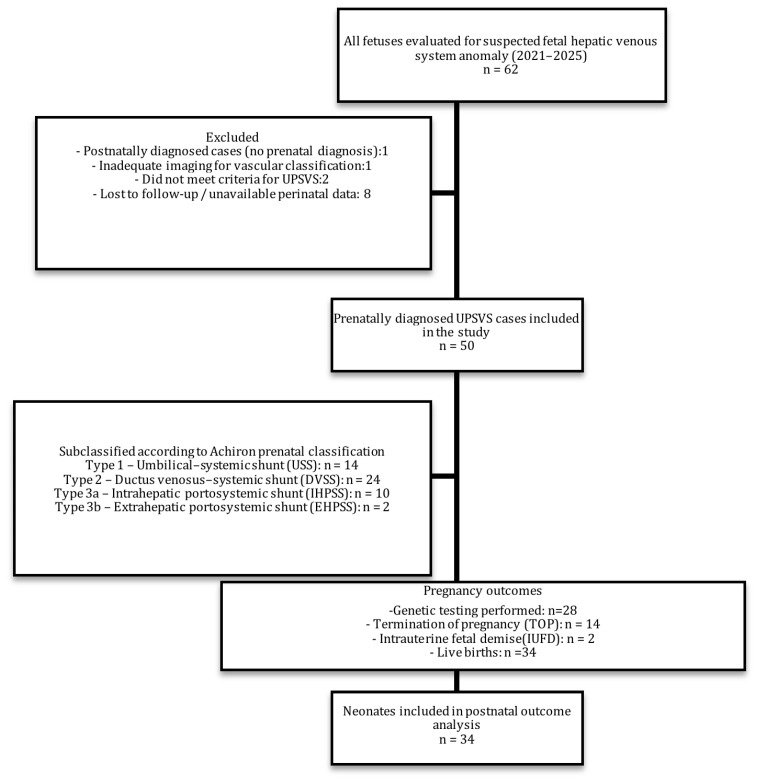
Study Flowchart of Case Selection.

The overall study design, case selection process, exclusion steps, prenatal diagnostic testing uptake, and perinatal outcome distribution are summarized in the study flowchart (Figure 7).

## 3. Results

A total of 50 fetuses with prenatally diagnosed UPSVS were included. Cases were classified according to the Achiron system as follows: Type 1 umbilical–systemic shunt (USS) in 14/50 (28%), Type 2 ductus venosus–systemic shunt (DVSS) in 24/50 (48%), Type 3a intrahepatic portosystemic shunt (IHPSS) in 10/50 (20%), and Type 3b extrahepatic portosystemic shunt (EHPSS) in 2/50 (4%). Baseline clinical characteristics and group distribution are summarized in Table 1.

Gestational age at diagnosis was significantly higher in the Type 3a group compared with Type 1 and Type 2 (32.2 ± 2.4 vs. 21.1 ± 6.7 and 22.4 ± 5.8 weeks, respectively; *p* < 0.001). Structural anomalies were most frequently observed in Type 1 fetuses, with a statistically significant difference across groups (*p* < 0.001).

Fetal growth restriction (FGR) was most prominent in Type 3a (IHPSS) fetuses, with 90% of cases affected, representing a significantly higher rate compared with other subtypes (*p* = 0.006).

Absence of the DV was universal in Type 1 (USS) and Type 3b (EHPSS), whereas no DV agenesis was identified in Type 2 (DVSS). In Type 3a, DV agenesis was observed in 20% of cases (*p* < 0.001).

Intrahepatic portal venous system (IHPVS) morphology differed significantly among subtypes. In Type 2 (DVSS) and Type 3a (IHPSS), the IHPVS was preserved in all cases (24/24 and 10/10, respectively). By contrast, in the Type 1 (USS) group, partial absence of the IHPVS was identified in 42.9% (6/14) and complete absence in 42.9% (6/14) of cases. Both Type 3b (EHPSS) cases demonstrated partial absence of the IHPVS (2/2) (*p* < 0.001).

Among fetuses undergoing invasive prenatal testing, genetic abnormalities were detected in 57% (8/14) of Type 1 and 56% (13/24) of Type 2 cases.

Intrauterine fetal demise occurred exclusively in the Type 1 subgroup (*n* = 2/14)

Termination of pregnancy (TOP) rates were highest in Type 1 (USS), with 57.1% of pregnancies electively terminated following prenatal diagnosis. A statistically significant difference in TOP rates was observed between Type 1 and Type 3a (*p* < 0.001). Structural and genetic abnormalities were the primary indications for TOP.

The distribution of genetic abnormalities together with associated structural anomaly status, including trisomy 21 rates, is presented in Table 2. Notably, in the Type 2 (DVSS) subgroup, 50% of structurally normal fetuses exhibited genetic abnormalities. In Type 3a (IHPSS), invasive testing uptake was limited, which should be considered when interpreting the absence of detected genetic abnormalities in this subgroup. When trisomy 21 was evaluated within this combined genetic–structural framework, the rates were 14.3% (2/14) in Type 1, 37.5% (9/24) in Type 2, 0% (0/10) in Type 3a, and 50% (1/2) in Type 3b (Table 2).

Associated structural anomalies were most frequent in Type 1 (USS), occurring in 92.9% of cases The detailed system-based distribution of associated anomalies is summarized in Table 3.

Among liveborn neonates, adverse postnatal outcomes were defined as ≥7 days of NICU admission or neonatal death after excluding secondary causes. Neonatal outcomes among the study groups are summarized in Table 1.

**Figure 8:**
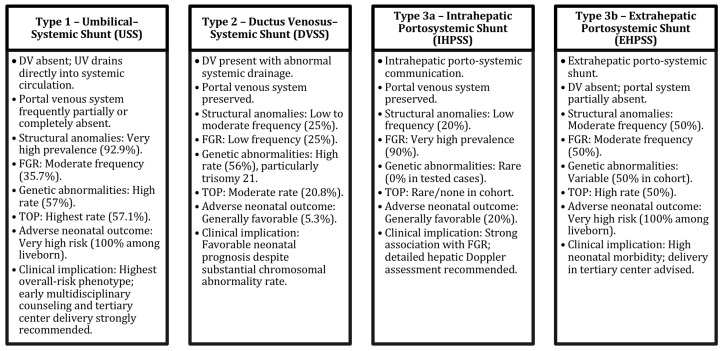
Conceptual Overview of Achiron Prenatal UPSVS Classification and Outcome Stratification.

Adverse outcomes occurred in 100% of Type 1 (3/3), 5.3% of Type 2 (1/19), 20% of Type 3a (2/10), and 100% of Type 3b (1/1) neonates (*p* = 0.002), indicating higher postnatal morbidity in Types 1 and 3b. In contrast, Type 2 (DVSS) demonstrated favorable neonatal outcomes. A schematic summary diagram integrating UPSVS subtype morphology with associated structural anomalies, genetic abnormalities, fetal growth restriction, termination of pregnancy, and adverse neonatal outcomes is presented in Figure 8. This conceptual overview visually synthesizes the outcome stratification patterns observed across Achiron prenatal classification subtypes.

## 4. Discussion

This study represents a relatively large single-center cohort of 50 fetuses diagnosed with UPSVS between 2021 and 2025, with systematic classification based on the Achiron prenatal scheme. The distribution of associated anomalies demonstrated that Type 1 (USS) fetuses exhibited the highest rate of structural abnormalities, highlighting that this subtype carries the greatest risk for multisystem involvement. This finding aligns with a previous series from Ankara, in which major and minor anomalies were more frequently reported in Type 1 cases [8]. In contrast, the lowest rate of associated anomalies in our cohort occurred in Type 3a (IHPSS) fetuses, a finding that differs from reports identifying Type 2 (DVSS) as the subtype least frequently associated with structural abnormalities [9].

In our cohort, DV agenesis was universal in Type 1 (USS) and absent in all Type 2 (DVSS) fetuses, underscoring the diagnostic value of DV assessment in differentiating UPSVS subtypes. This observation is consistent with Achiron’s seminal description, in which DV agenesis was present in all Type 1 cases and DV was preserved in all Type 2 cases [6]. The presence or absence of the DV thus represents a key component of the diagnostic algorithm and may serve as a differentiating criterion for proper subclassification.

The association between UPSVS and chromosomal abnormalities remains controversial and is still evolving. Some studies report high rates of chromosomal abnormalities, particularly trisomy 21, in association with UPSVS [10], while others have reported abnormal karyotypes in up to 40% of cases subjected to genetic analysis, irrespective of shunt subtype [9]. Conversely, certain reports describe considerably lower rates (approximately 9%) [6]. In the present study, genetic abnormalities were mainly concentrated in Type 1 (USS) and Type 2 (DVSS). This pattern is in line with larger cohorts reporting higher chromosomal abnormality rates in Type 1. When trisomy 21 alone was considered, the highest proportion occurred in Type 2 (DVSS), suggesting a possible predisposition toward trisomy 21 in this subtype, although the statistical power remains limited due to sample size. However, the relatively low rate of detected genetic abnormalities in the Type 3a subgroup should be interpreted with caution, as invasive prenatal genetic testing uptake was limited in this group. This variability in testing may have led to an underestimation of the true genetic association within Type 3a fetuses.

Type 3a intrahepatic portosystemic shunts are noteworthy for being diagnosed at later gestational ages compared with other shunt subtypes. This delay is thought to be primarily related to their deep intrahepatic location, small-caliber vascular connections, and the progressive maturation of the portal venous system, which becomes more conspicuous during the second half of pregnancy. As the intrahepatic portal vasculature develops and enlarges over time, abnormal communications may become more readily detectable on sonographic evaluation, thereby contributing to delayed prenatal recognition.

Another striking finding in our series was the strong association between Type 3a (IHPSS) and FGR. This association has been recurrently emphasized in recent studies, and several mechanisms have been proposed. Normal fetal growth requires adequate hepatic perfusion. In the presence of IHPSS, part of the oxygen-rich umbilical venous blood bypasses the hepatic parenchyma and enters the systemic circulation via hepatic veins, potentially reducing hepatic microcirculation and impacting fetal growth. Reduced hepatic perfusion may also decrease insulin-like growth factor-1 (IGF-1) levels, further contributing to FGR. Achiron similarly reported that fetal growth in Type 3a fetuses is frequently restricted and may be explained by reduced hepatic microcirculation, altered portal venous flow direction, and premature diversion of oxygenated blood into the systemic circulation [11,12]. Consistent with our findings, other studies have also highlighted lower birth weights in Type 3a fetuses [13]. In our cohort, 90% of Type 3a fetuses were affected by FGR, quantitatively reinforcing the proposed pathophysiologic link between reduced hepatic perfusion and impaired fetal growth. These observations suggest that detailed assessment of the hepatic venous system, including both afferent (Plan 1) and efferent (Plan 2) components, should be considered in fetuses with FGR to exclude an underlying IHPSS. Such an approach may enhance prognostic accuracy and guide perinatal management.

Postnatally, adverse neonatal outcomes were most common in Type 1 (USS) and Type 3b (EHPSS), paralleling findings from Li et al. (2025), who similarly reported high rates of adverse outcomes in Types 1 (85.7%) and 3b (66.7%) [14]. These results collectively support the view that Types 1 and 3b represent the clinically most vulnerable subgroups. Type 3a (IHPSS) generally demonstrated favorable neonatal outcomes; however, two neonates required prolonged NICU admission due to refractory hypoglycemia, elevated liver function tests, hyperammonemia, and hyperbilirubinemia. Notably, both fetuses displayed intrahepatic calcifications and hyperechogenic bowel prenatally, raising the possibility that intrahepatic calcifications may reflect localized ischemia secondary to reduced hepatic perfusion in the context of IHPSS, although data on this association remain limited.

Termination of pregnancy (TOP) decisions in our cohort were closely related to shunt subtype. As shown in Table 1, TOP was predominantly performed in the presence of major cardiac defects, multisystem structural anomalies, or abnormal karyotype. Type 1 (USS) exhibited the highest TOP rates, whereas Type 3a (IHPSS) rarely underwent TOP, reflecting marked differences in anatomic and hemodynamic characteristics between subtypes. These findings underscore the importance of early multidisciplinary counseling—particularly for Type 1—after prenatal diagnosis. In our center, TOP decisions were made following multidisciplinary consultation involving maternal–fetal medicine specialists, pediatric cardiologists, neonatologists, and when indicated, clinical geneticists. Cultural and legal frameworks governing termination practices may also have influenced decision-making patterns, particularly in structurally complex Type 1 cases. In contrast, Type 2 and Type 3a fetuses exhibited lower TOP rates, suggesting that shunt morphology and hepatic venous anatomy may play a significant role in prenatal prognostication. Although one study reported that all Type 3a cases underwent TOP, the authors attributed decisions to associated structural abnormalities in all three cases [15]. Overall, the literature suggests that TOP decisions are predominantly influenced by associated genetic abnormalities and major structural defects rather than the shunt itself.

Classification of UPSVS remains challenging, and discrepancies persist across the literature. Although the Achiron and Kivilevitch scheme remains the most widely used prenatal classification [6], inconsistencies arise due to varying emphasis on anatomic versus hemodynamic criteria. For instance, Wu et al. (2019) described a case morphologically resembling Type 1; however, the presence of a preserved DV with triphasic flow indicated hemodynamic features consistent with Type 2 [16]. Similarly, Erenel et al. reported six cases with preserved DV and triphasic flow consistent with Type 2 (DVSS) [17], yet these cases were listed as Type 1 (USS) in the Li et al. review [14]. Such discrepancies highlight the need for classification systems that integrate both morphologic and hemodynamic characteristics rather than relying solely on anatomy. Future classification efforts may benefit from algorithmic frameworks that combine shunt morphology, ductus venosus patency, waveform analysis, and portal venous system integrity into unified diagnostic flow models.

Finally, our findings indicate that Type 1 and Type 3b fetuses carry the highest risk of adverse neonatal outcomes, underscoring the importance of planned delivery in a tertiary center with neonatal intensive care support. Genetic abnormalities were most frequently encountered in Type 1 and Type 2 fetuses, and the relatively high frequency of trisomy 21 in Type 2 suggests that invasive prenatal genetic testing should be recommended when UPSVS is detected. The strong association between Type 3a (IHPSS) and FGR supports detailed evaluation of the hepatic venous system in growth-restricted fetuses. Systematic assessment of the DV, triphasic waveform, IHPVS integrity, and shunt morphology may improve classification consistency and enhance prenatal prognostication.

The strengths of this study include the relatively large sample size (*n* = 50), detailed subtype categorization, and postnatal follow-up. Several limitations should also be acknowledged. First, the retrospective design may introduce selection and information bias. Second, the uptake of invasive prenatal genetic testing varied across subgroups, potentially influencing the reported distribution of chromosomal abnormalities. Third, the number of Type 3b cases was limited, restricting subgroup-specific risk generalization. Finally, long-term neurodevelopmental follow-up data were not available, precluding assessment of extended developmental outcomes. Each of these factors may have influenced the interpretation of perinatal risk stratification.

## 5. Conclusions

UPSVS anomalies represent a challenging prenatal entity due to their heterogeneous embryologic origin, variable hemodynamic consequences, and broad spectrum of clinical outcomes. In this study, all UPSVS cases diagnosed in a tertiary center were systematically subclassified and evaluated in relation to associated structural and genetic abnormalities as well as postnatal outcomes, providing a meaningful contribution to the limited literature. Our findings indicate that the Achiron and Kivilevitch prenatal classification is clinically applicable and that shunt subtype is closely associated with perinatal course, suggesting that prenatal diagnosis may serve not only as a morphologic assessment but also as a prognostic tool.

The heterogeneous clinical behavior of UPSVS supports a subtype-based approach to prenatal counseling. Isolated forms generally demonstrate favorable neonatal outcomes, whereas the presence of major structural or chromosomal abnormalities warrants multidisciplinary decision-making, including the consideration of invasive genetic testing and perinatal management planning. Type 1 (USS) and Type 3b (EHPSS) represent the highest-risk subgroups for adverse neonatal outcomes and should be delivered in tertiary centers with access to neonatal intensive care. The strong association between Type 3a (IHPSS) and FGR highlights the importance of detailed hepatic venous system evaluation in growth-restricted fetuses.

Given the paucity of data correlating prenatal diagnosis with postnatal verification and clinical follow-up, this study underscores the need for future multicenter, prospective studies aimed at standardizing prenatal diagnostic algorithms, refining management protocols, clarifying the prognostic value of Doppler assessment, and optimizing genetic testing strategies. Prospective integration of morphologic, hemodynamic, and genetic parameters into unified classification frameworks may further enhance prognostic precision and clinical applicability.

## Figures and Tables

**Table 1:** Clinical characteristics of patients with anomalies of the umbilical–portal–systemic venous shunts (UPSVS).

Type	USS (Type 1) *n* = 14	DVSS (Type 2) *n* = 24	IHPSS (Type 3a) *n* = 10	EHPSS (Type 3b) *n* = 2	*p* Values	Post Hoc Comparisons *p* Values					
						Type 1 vs. Type 2	Type 1 vs. Type 3a	Type 1 vs. Type 3b	Type 2 vs. Type 3a	Type 2 vs. Type 3b	Type 3a vs. Type 3b
Maternal age (years)	26.6 ± 4.3	28.3 ± 5.4	28.4 ± 3.4	27.5 ± 2.1	0.727						
GA at diagnosis (weeks)	21 ± 6.7	22.4 ± 5.8	32.2 ± 2.4	23.2 ± 1.7	<0.001	0.999	<0.001	0.999	<0.001	0.999	0252
Structural anomaly on ultrasound	13 (92.9%)	6 (25%)	2 (20%)	1 (50%)	<0.001	<0.05	<0.05				
Genetic anomaly	4/7 (57%)	9/16 (56%)	0/3 (0%)	1/2 (50%)	0.335						
FGR	5 (35.7%)	6 (25%)	9 (90%)	1 (50%)	0.006		<0.05		<0.05		
DV absent	14 (100%)	0 (0%)	2 (20%)	2 (100%)	<0.001	<0.05	<0.05			<0.05	
IHPVS present	0 (0%)	24 (100%)	10 (100%)	0 (0%)	<0.001	<0.05	<0.05			<0.05	<0.05
IHPVS complete absent	6 (42.9%)	0 (0%)	0 (0%)	0 (0%)	<0.001	<0.05					
IHPVS partially absent	6 (42.9%)	0 (0%)	0 (0%)	2 (100%)	<0.001	<0.05				<0.05	<0.05
In utero demise	2 (14.3%)	0 (0%)	0 (0%)	0 (0%)	0.147						
Termination of pregnancy	8 (57.1%)	5 (20.8%)	0 (0%)	1 (50%)	0.012		<0.05				
Gestational age delivery (number of patients)	34.7 ± 6.5 (4)	37 ± 1.8 (19)	36.8 ± 1.5 (10)	37.4 (1)	0.463						
Birth weight (gram)	2426 ± 1239	2902 ± 538	2472 ± 582	3260	0.245						
Male	5 (35.7%)	14 (58.3%)	2 (20%)	0 (0%)	0.190						
Female	9 (64.3%)	10 (41.7%)	8 (80%)	2 (100%)	0.190						
Poor neonatal prognosis *	3/3 (100%)	1/19 (5.3%)	2/10 (20%)	1/1 (100%)	<0.001	<0.05				<0.05	

* Outcome rates in Type 1 and Type 3b should be interpreted with caution due to the limited number of liveborn cases in these subgroups. Abbreviations: USS—Umbilical–systemic shunt; DVSS—Ductus venosus–systemic shunt; IHPSS—Intrahepatic portosystemic shunt; EHPSS—Extrahepatic portosystemic shunt; DV—Ductus venosus; IHPVS—Intrahepatic portal venous system; FGR—Fetal growth restriction; GA—Gestational age.

**Table 2:** Genetic results of patients with umbilical–portal–systemic venous shunts (UPSVS) anomalies according to associated structural anomalies and the rate of trisomy 21 fetuses among groups.

Type	USS (Type 1) *n* = 7		DVSS (Type 2) *n* = 16		IHPSS (Type 3a) *n* = 3		EHPSS (Type 3b) *n* = 2		*p* Value
Genetic Anomaly									
	No	Yes	No	Yes	No	Yes	No	Yes	
No additional anomalies			5 (50%)	5 (50%)	3 (100%)	0	1 (100%)	0	0.211
Associated anoma-lies present	3 (42.9%)	4 (57.1%)	2 (33.3%)	4 (66.7%)	-	-	0	1 (100%)	0.696
Trisomy 21	1 (14.3%)		6 (37.5%)		-		1 (50%)		0.395

**Table 3:** System-Based Distribution of Associated Anomalies Among Fetuses with umbilical–portal–systemic venous shunts (UPSVS) Anomalies.

Associated Anomalies	USS (Type 1) *n* = 14	DVSS (Type 2) *n* = 24	IHPSS (Type 3a) *n* = 10	EHPSS (Type 3b) *n* = 2
Cardiovascular System				
Ventricular septal defect (VSD)	1	–	–	–
Atrioventricular septal defect (AVSD)	1	–	1	1
Tricuspid atresia	1	–	–	–
Hypoplastic left heart syndrome	1	–	–	–
Right aortic arch	–	2	–	–
Aberrant right subclavian artery (ARSA)	–	3	–	–
Total cardiovascular	4 (29%)	5 (21%)	1 (10%)	1 (50%)
Lymphatic Hydrops Spectrum				
Hydrops fetalis	1	–	–	–
Cystic hygroma	4	–	–	–
Total lymphatic	5 (36%)	0	0	0
Gastrointestinal System				
Duodenal atresia	–	1	–	–
Total gastrointestinal	0	1 (4%)	0	0
Genitourinary System				
Renal pelvic dilatation	–	2	–	–
Total genitourinary	0	2 (8%)	0	0
Musculoskeletal System				
Short femur	–	3	–	–
Total musculoskeletal	0	3 (13%)	0	0
Craniofacial System				
Nasal hypoplasia	–	1	–	–
Total craniofacial	0	1 (4%)	0	0
Other Structural Anomalies				
Single umbilical artery (isolated)	–	2	1	–
Diaphragmatic hernia	1	–	–	–
Liver calcification	1	1	2	–
Total other anomalies	2 (14%)	3 (13%)	3 (30%)	0
Multiple Congenital Anomalies				
Multiple congenital anomaly pattern	4	1	–	–
No Additional Anomaly				
None	1 (7%)	7 (29%)	7 (70%)	1 (50%)

## Data Availability

The data presented in this study are available on reasonable request from the corresponding author. The data are not publicly available due to privacy and ethical restrictions related to patient data.
